# AGE DIFFERENCES IN THE FREQUENCY AND NATURE OF ADULTS’ DAILY INTERACTIONS WITH OTHERS

**DOI:** 10.1093/geroni/igae098.2224

**Published:** 2024-12-31

**Authors:** Katherine Fiori, Amy Rauer, Kira Birditt, Angela Turkelson, Oliver Huxhold

**Affiliations:** Adelphi University, Garden City, New York, United States; University of Tennessee, Knoxville, Tennessee, United States; University of Michigan, Ann Arbor, Michigan, United States; University of Michigan, Ann Arbor, Michigan, United States; German Centre of Gerontology, Berlin, Berlin, Germany

## Abstract

Decades of research show that although adults’ social networks tend to shrink with age, the proportion of closest ties stays relatively constant and older adults rate their relationships as more positive. However, little research has examined with whom adults actually interact on a daily basis and the nature of those interactions. We used hierarchical network mapping data and ecological momentary assessment to examine whether there were age differences in social interaction types (inner circle, middle/outer circles, outside network) and in their valence (positive, negative, ambivalent), and if age moderated the link between interaction type and valence. Participants from the Stress and Well-being in Everyday Life (SWEL) study (N = 168; M age = 52.96, range = 33-91; 66.1% female; 47.6% Black) completed surveys every three hours for 4-5 days, reporting a total of 5,771 social interactions. Results showed that 36.6% of all interactions occurred with people outside of the network. Furthermore, against common theoretical expectations, only interactions within the inner circle declined with age. Moreover, although older participants reported more positive and more ambivalent interactions overall than younger adults, age did not moderate the association between interaction type and valence. This indicates that the increased positivity in older adults’ interactions was not specific to inner circle ties, but rather cut across interactions with all types of ties. Examining interactions on a daily level provides new insights into the specificity of potential age effects in social relationships.

